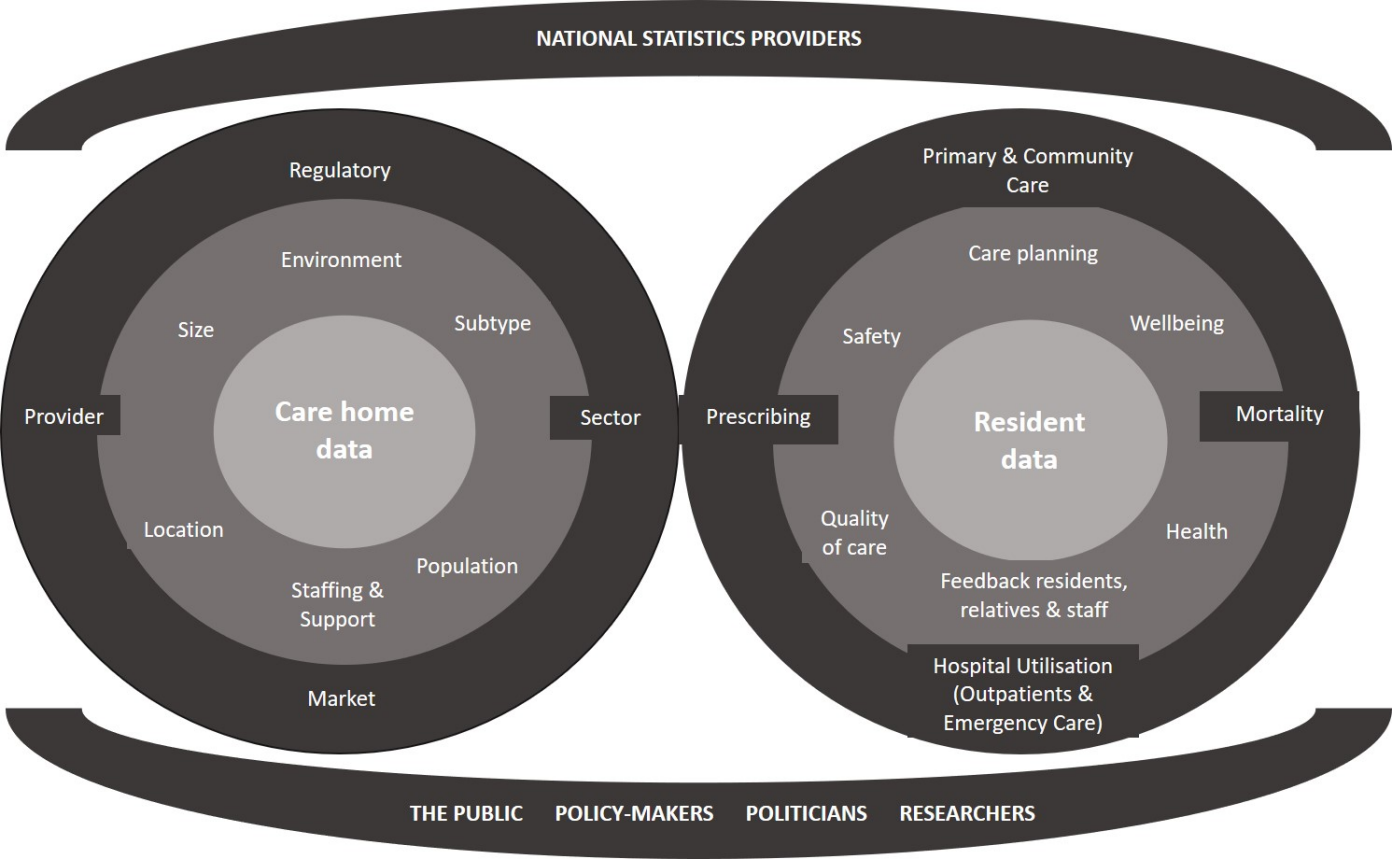# Closing the UK care home data gap – methodological challenges and solutions

**DOI:** 10.23889/ijpds.v5i4.1391

**Published:** 2021-05-12

**Authors:** JK Burton, C Goodman, B Guthrie, AL Gordon, B Hanratty, TJ Quinn

**Affiliations:** Academic Geriatric Medicine, Institute of Cardiovascular and Medical Sciences, University of Glasgow; Centre for Research in Public health and Community Care (CRIPACC), University of Hertfordshire; NIHR Applied Research Collaboration East of England; Advanced Care Research Centre, Usher Institute, College of Medicine and Veterinary Medicine, University of Edinburgh; Division of Medical Sciences and Graduate Entry Medicine, University of Nottingham; NIHR Applied Research Collaboration East Midlands; Population Health Sciences Institute, Newcastle University; NIHR Applied Research Collaboration North East and North Cumbria

## Abstract

UK care home residents are invisible in national datasets. The COVID-19 pandemic has exposed data failings that have hindered service development and research for years. Fundamental gaps, in terms of population and service demographics coupled with difficulties identifying the population in routine data are a significant limitation. These challenges are a key factor underpinning the failure to provide timely and responsive policy decisions to support care homes.

In this commentary we propose changes that could address this data gap, priorities include: (1) Reliable identification of care home residents and their tenure; (2) Common identifiers to facilitate linkage between data sources from different sectors; (3) Individual-level, anonymised data inclusive of mortality irrespective of where death occurs; (4) Investment in capacity for large-scale, anonymised linked data analysis within social care working in partnership with academics; (5) Recognition of the need for collaborative working to use novel data sources, working to understand their meaning and ensure correct interpretation; (6) Better integration of information governance, enabling safe access for legitimate analyses from all relevant sectors; (7) A core national dataset for care homes developed in collaboration with key stakeholders to support integrated care delivery, service planning, commissioning, policy and research.

Our suggestions are immediately actionable with political will and investment. We should seize this opportunity to capitalise on the spotlight the pandemic has thrown on the vulnerable populations living in care homes to invest in data-informed approaches to support care, evidence-based policy making and research.

## Introduction

The COVID-19 pandemic has had a devastating impact on UK care home residents, relatives and staff due to direct impact from the disease, and indirect impacts from isolation and changes to care provision [1]. Public, scientific and policy understanding of the pandemic has been hampered by the invisibility of care home residents in UK national data, which parallels wider stigmatisation and neglect of the sector [2].

COVID-19 has highlighted data failings that have hindered service development and research in care homes for years. Going forward, the priority is ensuring that health and social care data are fit for purpose in understanding care requirements and outcomes for care home residents more generally. This priority equally extends to other recipients of social care, including the housebound and those in supported accommodation and specialist housing, beyond the scope of this piece. This commentary summarises barriers to the effective use of care home data and discusses solutions to address this gap.

## What is a care home?

There is heterogeneity in the terminology used to describe care settings internationally [3]. In this commentary we use the term ‘care home’ which is an umbrella term to describe regulated care services providing 24-hour care to their residents. In some UK jurisdictions the terms residential and nursing home are used to differentiate, whereas others favour adult care home services. Data on the case mix and needs of residents within care home services are often lacking.

A key issue often overlooked is that UK care homes provide a home for adults of all ages, with specialist provision for those with learning disabilities, mental health problems, physical and sensory problems and substance misuse[4]. Although most care home residents are older adults, the population living in adult care home services is diverse and the needs of all groups living in care homes would be better understood with improved data collection. 

## Data Sources

Little is known about the UK care home population outside of research studies and even here they are excluded or under-represented in national cohorts[ 5]. National census data include resident-level data, but are usually neither timely [6], nor complete in coverage [7]. It is difficult to find basic demographic data, such as the number, age, sex and ethnicity of residents living in care homes. We also lack reliable normative data on care home length of stay and life expectancy.

It is not possible to readily identify the care home population within national data. During the pandemic, the denominator for mortality has been based on registered places, rather than occupancy. Production of care home-specific mortality has required data to be collated by the care regulators and shared with national statistics organisations. Consequently, where and when care home residents died remains unclear compared to recording of deaths in hospital. Aggregate mortality data became available later in the pandemic as the total number of residents dying compared to previous years’ deaths in care homes [8,9], but even this had limitations. Although English and Welsh data included in-hospital deaths of care home residents [9], Scottish data missed those who died in hospital. In summary, neither the numerator nor denominator for care home deaths during COVID-19 were accurate.

Such issues can be avoided if care home residency is identified across all population health, care and welfare datasets. A systematic, UK-wide approach to identifying care home residents in health and social care data could address this [10]. Local data solutions have been tested, but their use is not widespread [11,12]. Social care data sources based on funding status provide a partial account but miss those who fund their own care, who are not known by Local Authorities/Councils. 

## Flow between care settings

The pandemic has demonstrated the need to understand two issues; identifying who existing residents are; and tracking those admitted into or discharged from care home settings. Flow between hospitals and care homes was not well understood before the pandemic and it remains uncertain despite improved understanding of bed occupancy in some regions through central data collation. Usual activity in care homes is not systematically recorded or available at a national level, including information on short stays, intermediate care, respite provision and changes in long-term residency. It is these data, on the heterogeneity of provision and occupancy, that would support a greater understanding of the role that care homes play in how people move across the continuum of health and social care. Without this information it is impossible to evaluate the influence of short-term funding for local initiatives on the use of care home beds.

## Shared identifier to facilitate linkage

The challenge is that there are multiple sources of care home and resident data sitting in multiple unaligned databases, which are difficult to link because of a lack of shared identifiers. **Figure 1** summarises the multiple sources of care home data. Currently data linkage relies on probabilistic matching using variables including date of birth, name and address, which requires time and specialist skill (13,14). Linkage of care home data to other resources has facilitated useful research, for example by using care home admission as a long term outcome for randomised trial datasets [15]. However, probabilistic matching varies in accuracy between care homes due to varying data quality, and is not an ideal replacement for routine use of unique resident and service identifiers. At a resident level, NHS identifiers such as the Community Health Index Number in Scotland and NHS Number in England have not been commonly used in social care because they were perceived to bring no additional benefits to service providers.

Tangible benefits from data linkage can be demonstrated, for example, by linking resident and service variables so that case mix can be associated with changes over time in staffing level, skill-mix, bed capacity, service subtype and ownership. This, in turn, can enable decisions about organisation of care services and generate comparable national data. Examples of how linkage can drive service development comes from those countries which use care home minimum datasets [16]. A novel development is the potential to use the Unique Property Reference Number (UPRN), an Ordnance Survey unique numeric identifier for every addressable location[17]. This has the potential to identify care home locations as shared residences [18]. Routine use to enhance clinical practice and research will require specific effort to identify care home locations and investment to update address lookup systems and keep data systems contemporaneous with changes in services. 

## Individual level data to understand variation

National mortality data are reported at aggregate level [8,9] and regional analyses have been at population or care home service-level [19,20]. While these offer valuable insights, they cannot account for the heterogeneity in care home populations and the settings in which they receive care. Access to anonymised, individual-level data would enable more granular analysis of how variation in case-mix interacts with community infection prevalence and the impact of different service responses on outcomes. Since we don’t know who lives or works in care homes, we cannot examine how outbreaks and outcomes of infection are related to staffing skill-mix or resident case-mix, comorbidities and frailty.

## Analytical capacity

Care providers, local authorities/councils and staffing regulators collect and hold useful data, but mechanisms for secure data sharing are not established and organisations lack resource to prioritise data curation and analytics. The pandemic has placed challenges on the statutory regulatory role of the Care Quality Commission and the Care Inspectorates over care homes. They have had to use data to assure services, rather than relying on on-site inspection. Although data collection is an established part of their regulatory function, data have been shared and used for real-time monitoring during the pandemic in a way not previously seen. The reliance on the regulator for data preparation and curation highlights a wider lack of specialist analytical capacity within the social care sector. Investing in greater analytical capacity within the sector and collaboration with engaged academic institutions could enhance the capability to use routinely-collected data to inform practice and policy [21]. 

## Necessity of investing time to understand meaning

The focus for improving data quality and linkage to-date has been healthcare data [22]. The essential role of public involvement and engagement in data-intensive health research is recognised and promoted through consensus statement [23] and national campaign work [24]. Care homes are distinct settings from NHS facilities and the data collected there have their own purposes and meaning. Research using linked routinely-collected social care data requires investment of time, understanding the original data collection and interpretation, necessitating costed work with stakeholders [25]. This assumes a new way of working together with analytical teams in partnership with care home providers and other data controllers to interpret and contextualise the data. This approach to data integration can create meaningful, meta-data (e.g. codebooks and methods), which can be shared and made accessible for wider public benefit.

## Who controls access to care home data?

Access to anonymised NHS data is systematically managed by NHS Ethics Committees, Caldicott Guardians and the Scottish Public Benefit and Privacy Panel. Accelerated research permissions processes have been a positive development during the pandemic, facilitating timely data research in primary and secondary care [26,27]. There is no equivalent system of governance for care home data, which is currently held by a mixture of private companies, regulators, and health and social care providers. A particular challenge is in managing commercially sensitive data about the organisation and functioning of the sector [28]. There needs to be exploration of the acceptability of pooling information for the public good balanced against an organisations ability to provide care services as a business enterprise. In accordance with existing data governance processes [29], clear boundaries are needed about the purpose of data collection and acceptable reasons for sharing. For care homes, this will likely require delineation of data collected for a regulatory purpose, from data which can be shared with practitioners, researchers and government organisations. Experience within the NHS suggests that stakeholder engagement is likely to be effective as a mechanism for defining guiding principles and the acceptable boundaries for data sharing [30]. Developing integrated approaches to governance offers a way of utilising data that reflect this population’s experience of care, while ensuring that data can be accessed in a secure and safe way to the benefit of care recipients without compromising individual providers.

## Creating a core national dataset for care homes

Many of the issues raised above, about accessibility, governance, and ability to identify people as they move between services could be addressed by establishing a national core dataset based on resident-level information, linked to wider data sources. This would be underpinned by national minimum data standards, developed in conjunction with stakeholders to reflect the priorities of users. A dataset would need to be feasible, useful to frontline staff, and acceptable to residents and relatives, mindful of the burden of any novel data collection. Efforts to introduce internationally recognised tools, such as the Minimum Data Set, without a policy mandate have been unsuccessful [31] and studies have highlighted some of the implementation challenges that would need to be addressed going forward [32]. Principles of information governance could be built-in to protect the rights of residents, many of whom lack capacity to consent to data collection, collation and sharing. Such data could drive delivery of high-quality care and provide an analytical resource to explore variation within the population, support service development and enable relevant academic research. 

## Conclusions

The COVID-19 pandemic has exposed a critical knowledge gap for UK practitioners, researchers and policymakers, driven by the absence of high-quality routine data for one of the most vulnerable groups in society. It also provides the impetus to accelerate progress by investing in a data-informed health and care system. There is a critical need to understand the individual linked data sources and the context in which they have been collected. More effective utilisation and co-ordination of this data would be transformative in understanding the needs of this complex population, understanding pathways into care and the role care homes play in UK society.

## Acknowledgments

JKB is supported by a Scottish Clinical Research Excellence Development Scheme Lectureship, funded by NHS Education for Scotland. The funders played no part in the design or content of this article.

JKB, CG, ALG and BH are investigators on the Developing research resources and minimum dataset for care homes’ adoption and use (DACHA) study. DACHA is funded by the National Institute for Health Research (NIHR) Health Service Research and Delivery programme (HS&DR NIHR127234). Professors Goodman, Gordon and Hanratty are supported by the NIHR Applied Research Collaborations for East of England, East Midlands and North East and North Cumbria respectively. Professor Goodman also receives NIHR support as a NIHR Senior Investigator. The views expressed are those of the authors, and not necessarily those of the NIHR, NHS, or Department of Health and Social Care.

## Ethics statement

Ethical approval was not required for this Commentary article.

## Statement of Conflicts of Interest

The authors have no conflicts of interest.

## Figures and Tables

**Figure 1: The spectrum of care home and resident data available in the UK and key stakeholders:**